# Bursting out of our bubble: using creative techniques to communicate within the systematic review process and beyond

**DOI:** 10.1186/s13643-022-01935-2

**Published:** 2022-04-04

**Authors:** Jo Thompson Coon, Noreen Orr, Liz Shaw, Harriet Hunt, Ruth Garside, Michael Nunns, Alke Gröppel-Wegener, Becky Whear

**Affiliations:** 1 NIHR Applied Research Collaboration South West Peninsula (PenARC), University of Exeter Medical School, University of Exeter, Exeter, EX1 2LU UK; 2 University of Exeter Medical School, University of Exeter, Exeter, EX1 2LU UK; 3 University of Staffordshire, Stoke-on-Trent, UK

**Keywords:** Communication, Dissemination, Science communication

## Abstract

**Background:**

Increasing pressure to publicise research findings and generate impact, alongside an expectation from funding bodies to go beyond publication within academic journals, has generated interest in alternative methods of science communication.

Our aim is to describe our experience of using a variety of creative communication tools, reflect on their use in different situations, enhance learning and generate discussion within the systematic review community.

**Methods:**

Over the last 5 years, we have explored several creative communication tools within the systematic review process and beyond to extend dissemination beyond traditional academic mechanisms.

Central to our approach is the co-production of a communication plan with potential evidence users which facilitates (i) the identification of key messages for different audiences, (ii) discussion of appropriate tools to communicate key messages and (iii) exploration of avenues to share them. We aim to involve evidence users in the production of a variety of outputs for each research project cognisant of the many ways in which individuals engage with information.

**Results:**

Our experience has allowed us to develop an understanding of the benefits and challenges of a wide range of creative communication tools. For example, board games can be a fun way of learning, may flatten power hierarchies between researchers and research users and enable sharing of large amounts of complex information in a thought provoking way, but they are time and resource intensive both to produce and to engage with. Conversely, social media shareable content can be quick and easy to produce and to engage with but limited in the depth and complexity of shareable information.

**Discussion:**

It is widely recognised that most stakeholders do not have time to invest in reading large, complex documents; creative communication tools can be a used to improve accessibility of key messages. Furthermore, our experience has highlighted a range of additional benefits of embedding these techniques within our project processes e.g. opening up two-way conversations with end-users of research to discuss the implications of findings.

## Background

Increasing pressure to publicise research findings and generate impact, alongside an expectation from funding bodies to go beyond publication within academic journals has generated interest in using alternative methods of science communication to disseminate research findings. The UK National Institute for Health Research (NIHR) defines dissemination as ‘getting the findings of research to the people who can make use of them to maximise the benefit of the research without delay’ [1]. We know that most evidence users do not have time to invest in reading large and complex documents and may lack awareness or be unable to access newly published research [2, 3], and so in order to communicate effectively, we need to go beyond our academic ‘bubble’.

Over the last 5 years, we have explored a number of creative communication methods, to extend dissemination beyond traditional academic mechanisms and share our research with the diverse range of people who could benefit from the findings. Our general approach is based on evidence based guidance [4–7], principles of good dissemination [1, 8, 9] and examples of best practice relevant to the communication of systematic review findings [10–12].

Our aim in this paper is to (i) share and describe our experience of using a variety of creative communication tools (illustration, podcasts, infographics, blogs, briefing papers and board games and social media shareable content) and (ii) reflect on their use in different situations within the review process and thus to enhance learning and discussion amongst the systematic review community.

Underpinning our approach are three key questions:

i) What are the key messages?
ii) Who needs to know about them?
iii) How can we get the messages to the people who can use them?

Consideration of these questions will help guide decisions in involving evidence users in developing a dissemination plan that outlines key messages, identifies potential audiences and explores suitable format(s) and outlets for sharing messages which make the most of existing networks and opportunities.

At the heart of our work in this area is (i) an awareness of the need for producing different formats for different people, (ii) the importance of images to attract attention, and to aid learning and recall and (iii) a desire to communicate complex messages in easily accessible formats to foster enthusiasm and ultimately action. We are mindful of the need for evaluation to identify issues, learn from them and improve. To date, we have focussed primarily on informal formative and process evaluation as an ongoing process through consultation and feedback with representatives of those whom we hope will engage with our messages.

In the remainder of this paper, we provide examples of our experience in developing and using six communication tools for systematic review findings, illustrations, podcasts, blog posts, briefing documents, boards games and social media shareable content, before reflecting on our learning about creative communications.

### Illustration

Illustrations are drawings or images that give a visual explanation or interpretation of a topic or context. They have been used as a means of communication for centuries, adapted for different specialties and using a range of techniques. Today, illustration often sits under a broader concept of graphic communication whereby numerous design techniques can be brought together to communicate a topic in a variety of formats for different audiences.

‘…today the discipline of illustration is often lauded by the way it enriches and expands the lives, imaginations and sensibilities of individuals. In fact, it’s known to create and interpret cultural capital in all of its forms, most notably by its contribution to education and learning, research and new knowledge.’ Professor Alan Male (Emeritus Professor of Illustration, Falmouth University), Keynote address, International Conference on Illustration and Animation, 2015 [13].

We have used a range of illustrative techniques at several stages of the review process.

“Graphic Medicine” is a relatively new terminology coined to encapsulate the use of comics within healthcare for the purposes of education and patient care [14]. Comics can provide an opportunity for patients to share their experiences of medical care, treatment or living with an illness, which provides them with the opportunity to learn more about their illness and access support from peers living with a similar condition [14]. On the other side of the practitioner-patient relationship, comics also support practitioners to gain additional insight into the patient experience [15]. Comics, as a form of illustration, have the potential to create a shared narrative across different audiences and convey complex and/or emotion information in an engaging and accessible format [16]. We wanted to adopt the principles of this approach within the academic field to engage evidence users with the work that we do.

Scribing, sometimes known as Graphic Facilitation [17, 18], is a technique that uses a scribe to bring together key messages, ideas or insights from an event or meeting. Scribing usually involves the team working collaboratively with the scribe giving them as much information as possible beforehand for the context of the event, the intended aims, and what is expected of the output created. Scribing can take place in person or online, and with the scribe working on the illustration as the event is happening, it is possible to see progress immediately, dynamically reflect live events, actively engage attendees and make changes on the go.

Infographics aim to provide information and/or data in a form that is quick and easy for the audience to engage with and as such can use a variety of techniques to share information within the same output/product [19–24]. Infographics are often highly technical pieces of communication produced by trained professionals to simplify and ease communication of complex knowledge [24].

### Example 1: parent-to-parent support interventions for parents of babies cared for in a neonatal unit (PaReNt)

#### Stage of review: protocol development

This example is based upon a review evaluating evidence regarding the effectiveness and experiences of support groups for parents of infants admitted to neonatal wards [25]. We developed a *plain language protocol summary* (PLPS) which was intended to be a more accessible version of the scientific protocol [26]. Scientific protocols are usually written using technical language for the people conducting research. We wished to develop a more accessible version to support the parent advisory group to more fully engage with the review process by enhancing their understanding of the review rationale and processes, thus empowering them to share their knowledge and experiences.

Whilst the process of developing the PLPS helped us to identify the most important messages in a format which was definitely more concise than our full protocol, the final output was still well within our academic comfort zone. It was a three-page written document, and although it felt like an important step in improving communication from our perspective, our parent advisory group were nonplussed. On reflection, we felt we could do more to make the information accessible and useful. We decided to explore the potential for using illustrative techniques to convey the rationale, aims and methods of our project to parents and members of the public. Drawing upon the information provided within the PLPS, a researcher and artist from outside the project team (LS) created a series of illustrations to emphasise key points. These illustrations were then arranged in a series within a *one page document* through consultation with the Parent Advisory Group and project team.

#### Stage of review: dissemination

**Fig. 1:**
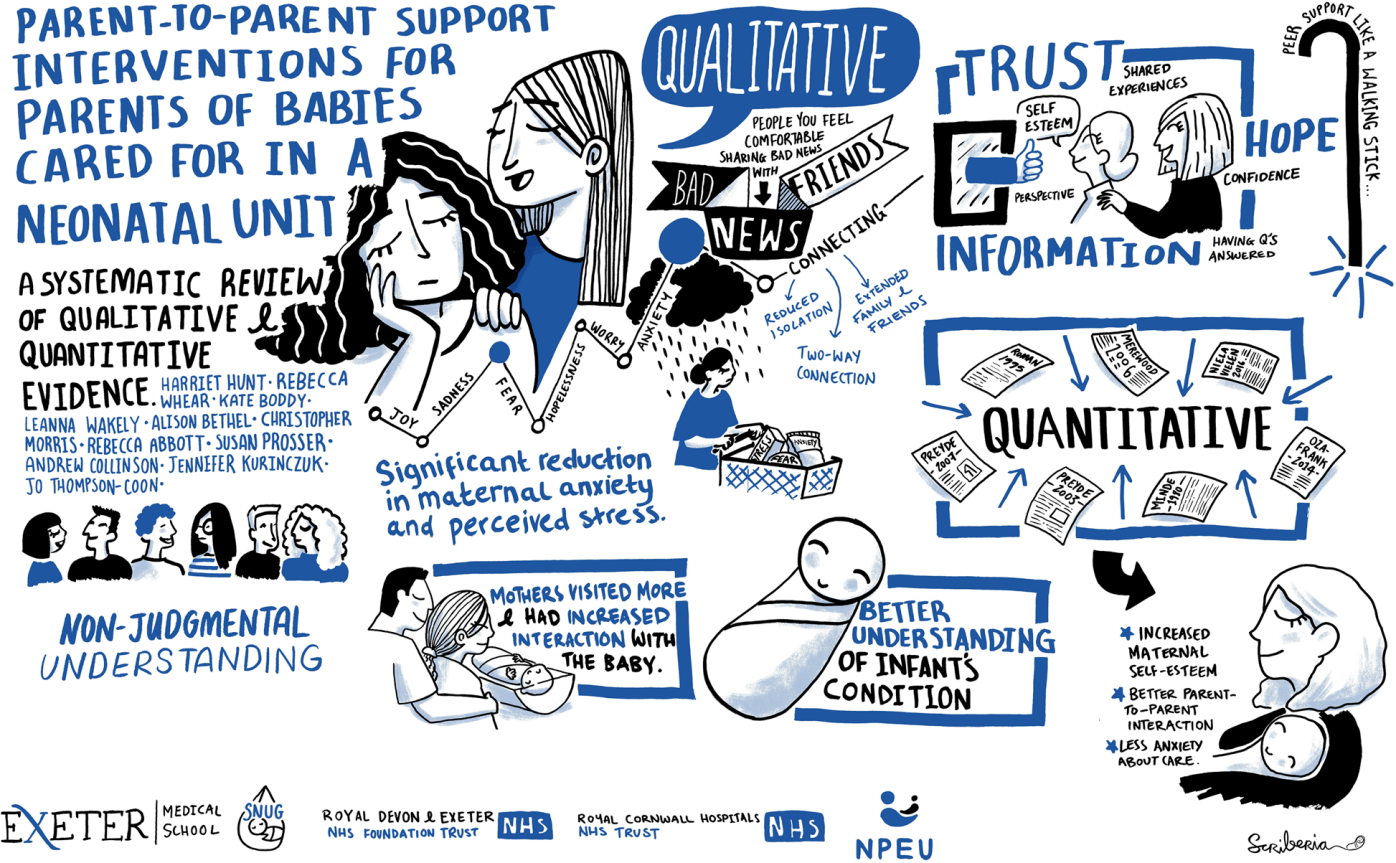
Infographic produced through live scribing: parent-to-parent support interventions for parents of babies cared for in a neonatal unit

We asked artist Rae Goddard from Scriberia (Scriberia.com) to attend and live draw two meetings where the results of the project were discussed, the final project meeting with the research team and the final meeting with the parent advisory group. Rae created a summary of key findings that evolved and developed during the meetings. The images captured a record of what was said including key quotes. We used the images to reflect on the parents’ experiences but also as mementoes to thank and acknowledge the parents for their contribution to the project. The final output (Fig. 1) involved interpreting both researcher and parent narratives to create a one-page visual summary of the project process, findings and implications for further clinical practice and research. This illustration was included within a newsletter for the BLISS charity, and postcards of the image were sent to neonatal units across the UK, along with an electronic image file with permission to be printed as posters for display on neonatal wards. Parts of the final image were also incorporated within a *Briefing Paper* summarising the review findings.

### Example 2: multicomponent hospital-led interventions to reduce hospital stay for older adults following elective surgery

#### Stage of review: dissemination

We used Graphic Medicine to support the dissemination of findings from a systematic review [27] aiming to evaluate the effectiveness and cost-effectiveness of interventions to reduce the length of stay for older adults admitted to hospital for planned procedures. A researcher within the project team (LS) created a series of illustrations to accompany brief text excerpts which summarised the rationale, methods, findings and implications of the review. We circulated the final *two-page document* to local patient groups and clinical contacts. The black and white format was intended to be suitable for photocopying and sharing within hospital waiting rooms in a way which minimised use of hospital resources. We also incorporated individual images into conference posters and the project *Briefing Paper* for clinicians and service commissioners. Due to time-limitations, it was necessary to focus on producing one comic which could be shared with a variety of stakeholder groups. We would like to use the images to create materials which identify and communicate messages tailored to the specific needs of different groups to enhance the project’s accessibility and relevance to different stakeholders.

### Example 3: improving continence in children and young people with neurodisability

#### Stage of review: interpretation of findings; dissemination

**Fig. 2:**
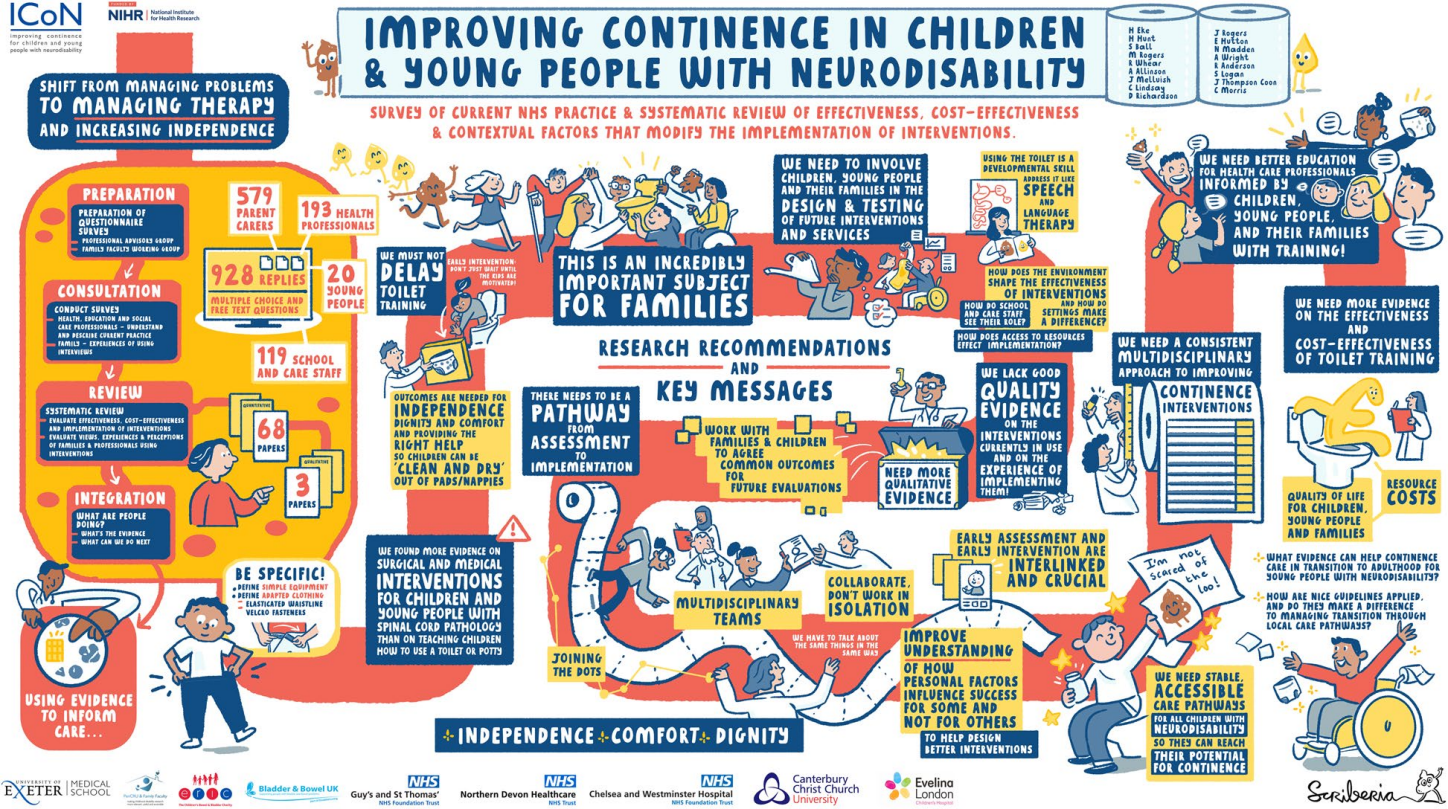
Infographic produced through live scribing: improving continence in children and young people with neurodisability

In this example, we used digital scribing undertaken during virtual meetings, to bring together the findings from a survey and a systematic review of interventions to improve continence in children and young people with neurodisability [28] (Fig. 2). This project involved a large number of stakeholders, e.g. clinicians involved in the care of children with neurodisability, representatives from third sector organisations and parents, and concluded during the early months of the COVID-19 pandemic. We had planned to hold a whole team face to face meeting and present and discuss the findings with an artist present but it became clear that that would not be possible due to pandemic restrictions. We therefore discussed and agreed the key findings as a team before sending a summary to the artist, Matt Swan of Scriberia (Scriberia.com). One member of the research team then met with the artist virtually to discuss the project and the key findings. Matt produced a mock-up of the eventual illustration before attending a virtual team meeting where we discussed the findings in more detail to produce a final agreed version of the infographic.

### Example 4: using creative techniques to communicate systematic reviews

#### Stage of review: dissemination

**Fig. 3:**
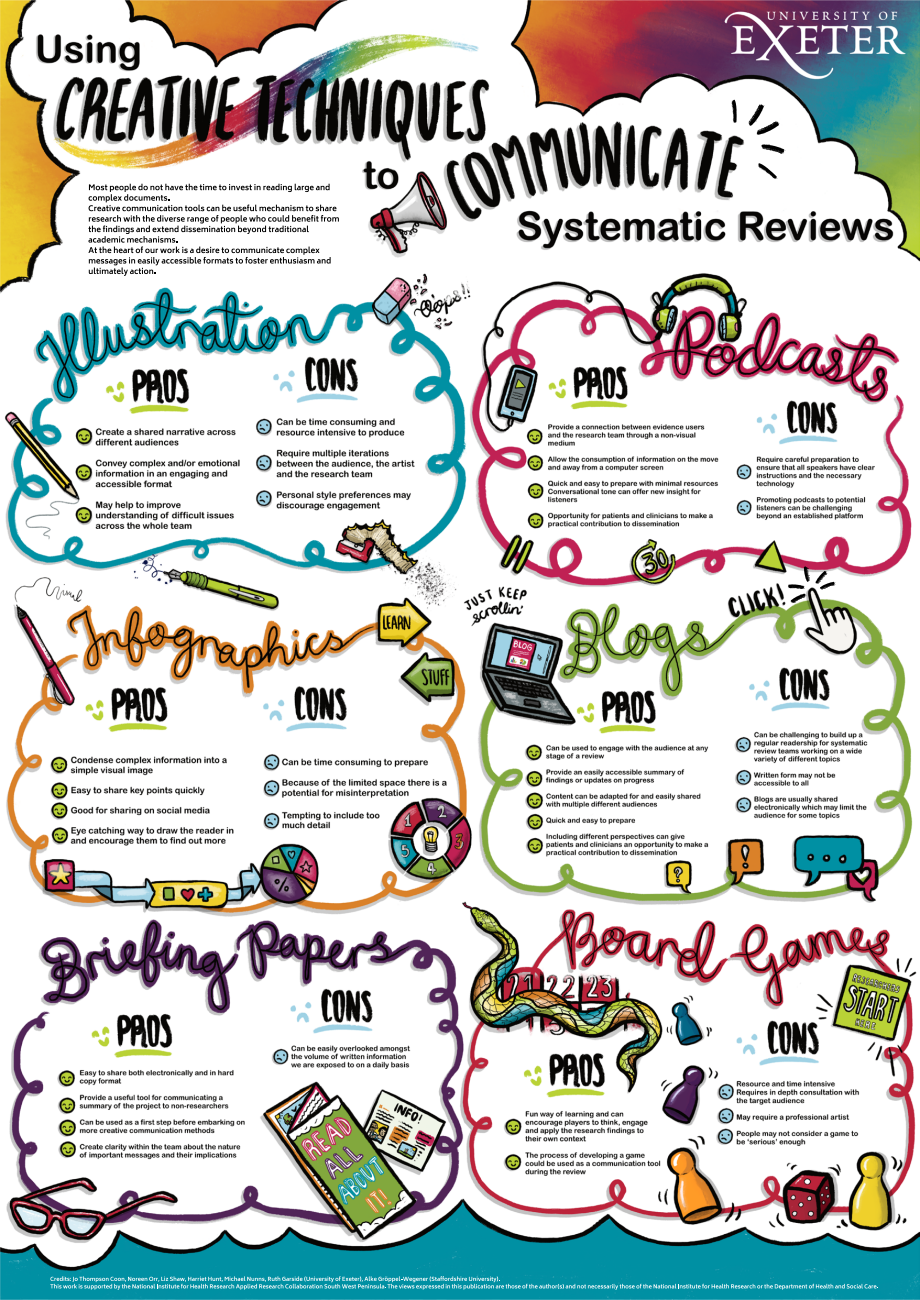
Infographic describing the use of creative techniques to communicate systematic reviews

In this final example, we worked with a sketch note artist, Grace Elizabeth, to create an infographic (Fig. 3) that describes the various methods of creative communication discussed within this paper and the advantages and disadvantages of using them. Development of the infographic involved an iterative process of discussing and sharing key points with the artist and editing and amending draft illustrations. We also shared draft versions of the infographic with individuals unconnected with the paper to check external understanding. The infographic was used to present our work at a national conference. Discrete sections of the infographic have also been used on social media.

### Reflections on using illustration

Overall, illustration can be an interesting and engaging method to involve key stakeholders with both the process of conducting systematic reviews and sharing the findings. Whilst producing illustrations can be labour intensive and require additional time (if utilising in-house expertise), or financial resources (if hiring a professional artist), we have found that the outputs can be used flexibly across a range of academic resources and can be an easy and cost-effective method to share project information and findings with evidence users. To maximise the usefulness of this type of output, we recommend the final illustration represents collaboration and a shared understanding between researchers, evidence users and artist.

### Podcasts

Podcasts offer the opportunity to condense the information presented in a fairly lengthy document into a few minutes of audio, whilst adding a conversational tone and connecting with evidence users through a non-visual medium [29]. Podcasts also broaden accessibility and allow the consumption of information on the move and without looking at a computer screen [30].

### Example 1: interventions to improve the mental health of young people with long-term conditions

#### Stage of review: dissemination

We produced several podcasts as part of the dissemination of the findings from a complex systematic review of interventions to improve the mental health of young people with long-term conditions [31]. We initially recorded two podcasts: one to allow members of the research team (researchers, clinicians, young people and their parents) to describe the findings of the systematic review and one for researchers, young people and their parents to reflect on the process of involvement in the project.

These podcasts were resourced by the dissemination budget allocated to the project. We were able to travel to London to record conversations with the young people involved in the project and their parents. We also had the support of a science communications specialist with professional recording equipment who guided us through the process, from developing the script to producing good-quality audio and editing the audio clips. This support was invaluable in producing a seamless and professional product from multiple recording sessions.

The finished products are hosted on our *project page*, are available on *SoundCloud* and were shared via Twitter alongside the release of the final report. Our discussions with the young people and their parents indicate that producing the podcasts was a rewarding experience not least because it provided an opportunity for their voices to be heard on a subject that they felt strongly about. They also enjoyed being able to make a practical contribution to the dissemination of the research.

A further *podcast* about the project was recorded and featured alongside a blog by the ‘Mental Elf’ (@MentalElf) following the 2018 Cochrane Colloquium in Edinburgh, UK. This involved Erin Walker, Patient and Public Involvement Lead, and Katrina Brooks, a young person from our advisory group, discussing their experiences of being involved in the project.

### Example 2: Exeter HS&DR Evidence Synthesis Centre

#### Stage of review: dissemination

Having previously produced a successful set of podcasts, we were aware of the value of such an approach. However, we found it to be a fairly time-consuming and resource-heavy endeavour, requiring recording equipment, multiple meetings and technical expertise, which we considered prohibitive. That was until we were inspired to pilot a regular research podcast by a twitter discussion, when a member of the public professed that it would be nice to hear academics talk about their work. We wanted to know whether a podcast could effectively confer the findings of a research study and whether it would be achievable with a low-tech, low-resource solution. We found that this was achievable and have now produced two podcast episodes to accompany outputs produced by the Exeter HS&DR Evidence Synthesis Centre.

The first described the findings of a rapid review of evidence to inform the Independent Review of the Mental Health Act (1983) in late 2018, with a single speaker describing the work. This was quick and easy to do, using a podcast producing app called Anchor on a smartphone. Anchor allows audio to be recorded and re-recorded with the touch of a button, in the comfort of the office or home, without the requirement of additional equipment (although this can be added to enhance quality).

For the second episode, we described the findings of a large review of interventions aiming to reduce length of stay in hospital for older adults undergoing planned surgery. Rather than a single speaker, we wanted to include a range of voices including clinical stakeholders and members of the review team.

In order for this to run smoothly, we needed to ensure that all speakers had clear instructions about their contribution to the overall episode, had access to the required technology (smartphone) and could contribute with minimal time commitment. By combining separate recordings we were able to have one researcher ‘hosting’ the podcast, asking questions of the other contributors and varying the delivery of the episode. We promoted links to the podcast on social media alongside the final report and links are available on our *project pages*.

### Reflections on using podcasts

If done well, podcasts can engage the audience, allowing easy access to key messages and a connection with members of the research team [32]. Access to research findings is enhanced by providing an alternative to visual dissemination materials, and the conversational tone of podcasts may allow the audience new insight into the research [29]. We found it was possible to produce adequate quality podcast episodes with ease and were able to combine the perspectives of various contributors remotely. In our first example, it was particularly rewarding for young people and their parents to be actively involved in producing dissemination materials about research in which they have been involved and were passionate about. In our experience, podcasts can be recorded in a variety of settings, without access to a computer or professional recording equipment. We have found that without regular content to promote the podcast, numbers of listeners can be relatively low. For example the podcast hosted on the established ‘Mental Elf’ blog has been listened to over 400 times, whilst those hosted on our relatively unfrequented research pages have had 40–50 listens each. If choosing to capture audio from a group discussion, or when looking to produce higher quality audio, additional recording equipment may be required. It may also be time-consuming to record if several re-takes are needed or interruptions take place. Time to become familiar with the software and some degree of training may be required to allow contributors to record clips remotely, although this can be done quite quickly. It can also be helpful to have a ‘non-academic’ listen to the final podcast prior to publication, to ensure its tone and language is interesting and engaging. Finally, a podcast series is likely to be more effective and to build up a listener base with regular episodes, but it can be difficult to keep the momentum going—particularly if waiting for the end of a project to produce the next episode.

### Blog posts

Blog posts or short, written, accessible opinion pieces can be used to talk about a systematic review using a relaxed but informative and engaging style often from a variety of perspectives [33, 34]. This approach to communicating can be adapted for most audiences [35] and can be used to talk about a review at any stage, for example, experiences in developing the protocol with stakeholders, or views from researchers on working on a systematic review for the first time. Producing regular blog posts can be a helpful mechanism to build interest in a review before it is published, and it can also be informative for those interested to understand methodological challenges.

### Example 1: blog series on the perspectives of patient-initiated appointment systems for people with long*-*term or chronic conditions

#### Stage of review: dissemination

In 2020, we published a Cochrane systematic review [36] that looked at the use of patient-initiated appointment systems in place of consultant-led appointment systems for people with chronic and recurrent conditions being managed in secondary care (hospital outpatients department). The review itself had taken some time to finally bring together and publish and during that time the idea of patient-initiated clinics was becoming increasingly talked about. We knew that the review findings would be well publicised in various forms with the help of the Cochrane team, and we had also published a briefing paper on the review to increase accessibility for a wider audience. So, for the blog series we decided to focus on the intervention itself and reflect on how it was received by a range of stakeholders: a *service-user*, a *clinical nurse specialist*, a *consultant*, a *service manager* and a *health service commissioner*). Whilst this could have been incorporated into one post, it would have been very long so we decided to make a blog series—one post per perspective—that could be published over a number of days or weeks. An additional advantage of this approach was that each new post not only directed readers to the review (and associated dissemination outputs) but also to the other blog posts in the series, those that had already been published or those that were forthcoming. We created tweets to share the blog posts to a wider audience and also asked our stakeholders to share the links. The series was well received in terms of comments and re-tweets.

### Example 2: a single blog post on social work practice in the UK

#### Stage of review: scoping

In 2017, our Exeter Health Services and Delivery Evidence Synthesis Centre was commissioned by the HS&DR programme to undertake a systematic review of the effectiveness of different models of social work practice in the UK. Our first task was to become familiar with the area and to explore the available evidence. In doing this, we realised that there was very little evidence available that could be synthesised and the project did not progress. We felt, however, that a summary of our scoping of the topic might be a useful resource for others and could also provide some insight into the processes that we use to become familiar with a topic. We posted the *blog post* on our team blog—*Sifting and Sensemaking*—in July 2017 and shared the link via both our Twitter feed and via email to those who had been involved in the scoping process. The blog post was well received in terms of comments and re-tweets and allowed the review team to feel that although the project was paused due to lack of available evidence, their work in reaching that decision could still be useful.

### Reflections on using blogs

Blogs can be used in lots of different ways and at different stages of the review process to engage with the audience. We have found that preparing blog posts, especially with other stakeholders, can help to clarify key messages, provide an outlet for discussion and increase the diversity of people who engage with review findings. However, it can be time consuming in terms of identifying and liaising with the contributors, preparing the posts on the blog site, finding the appropriate images and devising tweets and emails to promote them. Publishing the methods and outcomes during the review process can help to improve the transparency of the work undertaken by the team, something which is important to establish trust between scientists and evidence users.

### Briefing papers

Short, plain language summaries of systematic reviews are an increasingly common dissemination output [9]. In keeping with the underlying purpose of the approaches discussed in this paper, we define briefing papers as short, accessible, engaging summaries of systematic review findings designed to go beyond an academic audience. Guidance on preparing briefing papers for policy makers suggests a 1:3:25 page format with one page of take home messages, a further 3 pages that describe the methods, findings and implications in more detail and a full (25 page) report [37].

### Example 1: Exeter HS&DR Evidence Synthesis Centre: the Briefing

#### Stage of review: dissemination

For this *programme of work*, we have developed a template that combines the 1- and 3-page summary to create a *4-page document*, tailored to the anticipated audience and focused on the findings and implications with little methodological detail. The front page presents a complete summary of the review with key take home messages and the following pages provide explanation of context, brief methods, findings and implications for practice, policy and research. We have also used this template in a number of other projects and found it to be easily adaptable to different review topics and audiences.

### Example 2: how do “robopets” impact the health and well-being of residents in care homes?

#### Stage of review: dissemination

For this review, we created a newspaper style *briefing paper*, *The Woofington Post*, to share our findings with care home staff. Methodological detail was minimised to dedicate space to communicating key messages arising from the research. Based on our experience of working with care home staff, our intention was to produce a short, easy to read and visually appealing briefing paper that would be suitable for staff to read on breaks. We shared this briefing paper predominantly in hard copy format in response to feedback that many care home staff do not spend their breaks in front of computers or devices. *The Woofington Post* is also available electronically and the visually appealing format has proved popular on Twitter.

In both examples, we provide easily accessible links to signpost the reader to the full report, other related resources and the contact details for the research team and thereby provide further information for those who are keen to have a more in depth understanding of the research.

### Reflections on using briefing papers

Briefing papers often form the mainstay of our communication activities as they provide a quick summary that is easy to share both electronically and in hard copy format. We have found them to be a useful device in communicating with, for example, creative colleagues to inform discussions about producing other outputs and, with support groups and third sector organisations to facilitate communication and enable effective decision making about the suitability of the research for newsletter or website pieces. The process of creating briefing papers itself can also be beneficial in creating clarity within the team about the nature of the important messages and their implications. The written form may not be accessible to all and this type of output can be easily overlooked amongst the volume of written/electronic information we are exposed to on a daily basis.

### Board games

The well-known concept of a board game is a fun way of engaging people with many subjects. For example, educational board games help learners increase knowledge and stimulate discussion [38]. As an effective way of breaking down complex information into an easily accessible and enjoyable format, there is potential for board games to be used to explain research processes and research findings [39]. This works on two different levels: as a visual metaphor or at the level of game mechanics. The former uses visual clues to evoke the concept of the board game, for example the idea of a game path can dress up a clinical looking flow-chart and immediately communicate the notion of progress, as well as detours and short-cuts. This is a variation of an illustration but uses familiar board game aesthetics to evoke a playful genre (and possibly mood for the audience). It allows the most important pieces of information to be embedded within the design of the ‘board’. The second level is not just about the visuals, i.e. it is possible to make some project outcomes look like a board game at the same time as building in game mechanics to make them playable. This raises the engagement factor of the communication—as members of the audience become players they might experience the frustration of encountering a chance card triggering an unfortunate event, for example. It is also possible to add information to and through gameplay and rules—for example have different rules for different demographics to highlight special risk factors of those demographics.

### Example 1: Pet Purrsuit—a board game to share evidence on the impact of animals in care homes

#### Stage of review: dissemination

**Fig. 4:**
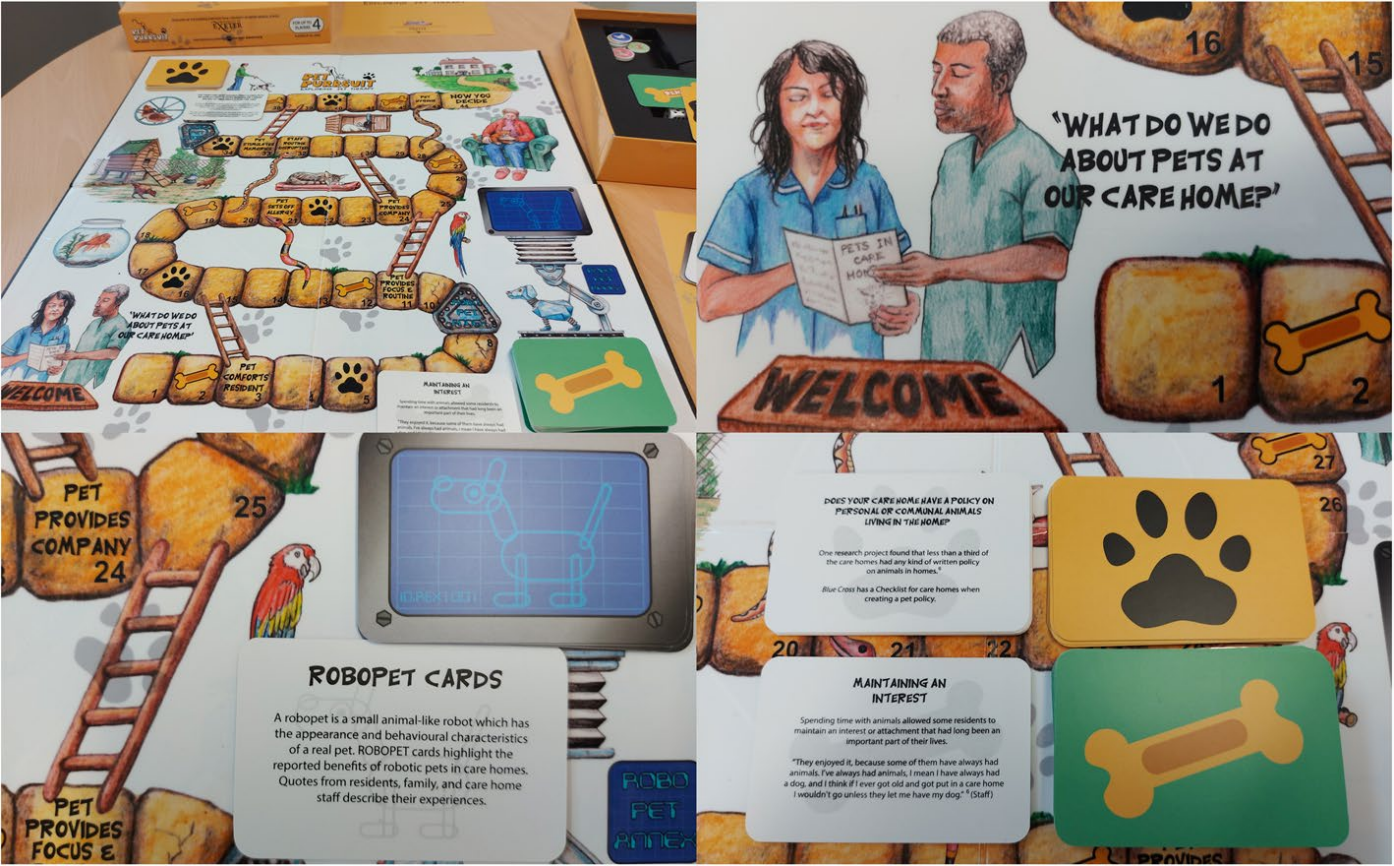
Pet Purrsuit board game

We developed a board game, Pet Purrsuit (Fig. 4), to share evidence from two systematic reviews on the impact of animals and robotic pets on the health and wellbeing of older people living in care homes (paper in preparation [40];), in order to engage care home staff with our research findings. Our aim was to design a game that would help care home staff consider the benefits and challenges of having animals reside in, or visit their care home. We followed Groppel-Wegener’s three plus one stages in board game design: diagram stage, game mechanics, visual impact and assembly [41]. In the diagram stage, which drafts the game path, we focused on the decision-making process for staff considering introducing animals to the care home. In the game mechanics stage, we discussed different ways of introducing potential shortcuts and obstacles and triggering chance events (for example, game cards). We devised two types of game cards, ‘treats’ cards which would give the player a turn and ‘paws’ cards which would mean that the player missed a turn. The ‘treats’ cards highlight some of the reported benefits of animal therapy, whilst the ‘paws’ cards consider issues such as staff training and the practicalities of having resident/visiting animals in the care home. During the visual impact stage, we considered potential visuals to illustrate the content of the game and thought about how we could organise the game path. The game path follows the world wide classic ‘snakes and ladders’ board with numbered, gridded squares, some of which were allocated to ‘treats’ and ‘paws’. We sketched out ideas that included animals that are often found in care homes and ways in which we could incorporate a ‘residential home’ into the design. Lastly, in the assembly stage, we brought together the previous stages with a sketch of the prototype board game.

We then worked with a local artist and designer to design and create a visually appealing and professional looking prototype board game, Pet Purrsuit which incorporates images of care home staff, residents and animals.

Initial observations from playing the game with researchers and care home staff suggest that the board game is an engaging way of learning which encouraged the players to think and engage and apply the research findings to their own context. Conversations occurred during the gameplay, as the players read the content of the ‘treats’ and ‘paws’ cards aloud. Several aspects of our approach to designing the game helped make it easy for people to engage including basing the visual appearance and the rules on familiar games and permitting play to stop and resume later. We believe that playing the game may also have the potential to flatten the power hierarchy between researchers and research users.

### Reflections on using board games

In this example, the board game was used for dissemination but this method of communication could provide an opportunity to engage with stakeholders in other stages of the review process, e.g. in explaining the scope and remit of a review albeit this would require a less polished product. Researchers should note that designing and developing a board game is resource and time intensive, in this case, requiring a professional artist and consultation with stakeholders. There is also the possibility that the concept of a board game as a way of engaging with research may be viewed by some as trivialising the research and engagement may be limited if playing the game requires facilitation. People are motivated to play games for different reasons [42], and it may be a challenge to develop a game which appeals to all audiences. As a result of the global coronavirus pandemic, we have had limited opportunities to explore these issues in depth with care home staff.

### Social media shareable content

Social media shareable content is content that can easily be shared on social media as a means of signposting the audience to more substantial outputs. Using visual images has been shown to be effective in increasing engagement with social media [43–45] which is perhaps not surprising given that the use of images to improve attention to and recall of information is a well-known approach in health education [46]. The use of visual snapshots of the findings of systematic reviews is a technique frequently used to share Cochrane evidence [47] in a dissemination product they refer to as blogshots.

### Example 1: World Alzheimer’s Day

#### Stage of review: dissemination

**Fig. 5:**
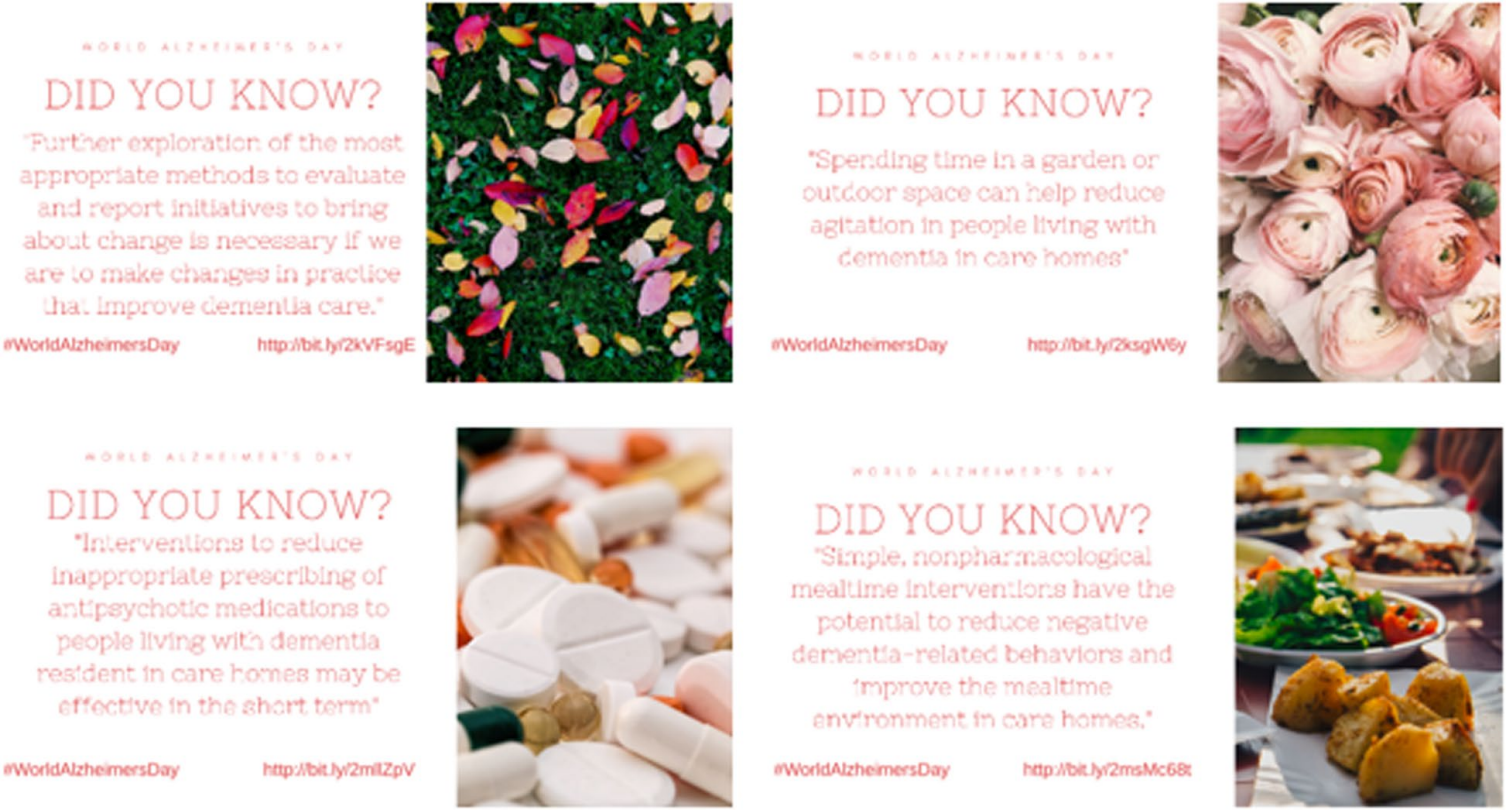
Examples of social media shareable content created for World Alzheimer’s Day

We recently created and shared a series of Twitter cards in order to share our relevant research in celebration of World Alzheimer’s Day (Fig. 5). Each card has a similar design and contains a short statement of the main findings from the review, a visually appealing image and a link to the published paper. We shared the cards on Twitter over the course of the day.

### Reflections on using social media shareable content

Social media shareable content is relatively quick to create using existing content. In this example, we were able to highlight a collection of previously published research relevant to World Alzheimer’s Day in a cohesive style. This form of dissemination enables the sharing of key messages from a published paper in an eye-catching format. As this example demonstrates, it can also be used to promote previously published research (rather than new outputs) as part of a themed collection. It can, however, be challenging to summarise the key points with the necessary degree of nuance in the space available.

## Discussion

In this paper, we have described our experience of using a variety of creative communication tools at different stages of review production and dissemination and reflected on the advantages and disadvantages of each tool. Our reflections are captured in an infographic (Fig. 3). Collating our combined experiences has given us an opportunity to reflect on some of our shared learning which we have organised into seven key areas: the aim and purpose of creative communication products, benefits of a multi-layered approach, consideration of digital vs. non-digital outputs, the role of non-researchers, choice of images, evaluation and unexpected benefits.

### Aim and purpose

In common with many others [6, 48–50], the underlying aim and purpose of our creative communication products is to present key messages in useful and accessible ways to improve engagement with our work. Whilst this may feel self-evident, we have found that it is useful to actively reflect on the aim and purpose and to remember that any creative communication product is unlikely to be able to present the whole story. It can be challenging to identify and distil the important information for a lay audience from the wealth of detail required by academic audiences. Working closely with non-researchers to understand the level of useful detail is helpful, as is being prepared to work iteratively and to make numerous changes. It can be helpful to allow time to reflect on whether sufficient information is provided to allow the reader to apply the appropriate level of confidence to the findings. Sharing with researchers who are not closely involved with a project and asking them to feedback what they understand from a creative communication product can be invaluable.

### Multi-layered approach

As others have described [49, 51], we have often used a multi-layered approach to communication. For example, producing a briefing paper (which includes illustrations created specifically for the project), a blog and a podcast and sharing these outputs by social media as well as via more traditional methods, e.g. mail outs. With their STEP tool, Elijz and colleagues [52] plot the relationship between complexity of the information to be communicated and the available level of interaction with potential audiences. We (as others) would argue that even with this in mind, one size is unlikely to fit all, and at different times, individuals will have different preferences for the balance between complexity and interaction and between words, graphs, images and videos. Hence, by linking together the various products to enable audiences to access some or all of them, our intention is that the information we seek to share is accessible to individuals with a range of engagement preferences [49, 53]. Furthermore, sharing key messages in bite sized packages exploits the audience’s desire to find out more—the ‘pull’ model of knowledge translation [54].

### Digital vs. non-digital

Getting to know your audience and their preferences for accessing information is an underlying principle of effective science communication [55, 56]. By working closely with the people who we hope will use the findings of our research in their practice, we have learnt not to assume that a digital output via email or social media will be the most convenient format for audiences. Unlike academics, many health and social care professionals do not spend the majority of their time in front of screens and a paper version of a creative communication product may be more useful and relevant. For example, after discussion with care home staff, we chose to share the briefing paper for our robotic pets review [40] as a paper document as staff felt they were more likely to pick something like this up and read it in their break than open an email after work. Likewise, following a request from staff, we offered the illustrations created during our peer support in neonatal units project [25] as a poster for display on the ward and created postcards with the illustrations on one side and further details on the other .

### Role of non-researchers

In addition to working with representatives of the potential audiences during the review process, non-researchers have had an essential role in directly or indirectly shaping all of our creative communication products. Starting early, allowing sufficient time to distil the key messages for different audiences, developing a plan and engaging the right people to deliver the plan are all important elements of effective communication [10]. We have found non-researchers particularly helpful in de-prioritising the methodological detail that as researchers we are keen to share, in preference to promoting messages which will be influential in contributing to changing practice or behaviour.

### Choice of images

Just as images can be powerful in engaging a potential audience, the wrong choice of image can prevent effective engagement and draw attention to a creative communication output for the wrong reasons [57]. An example is the use of images of wrinkly hands to share stories about older people on social media which became associated with the hashtag #NoMoreWrinklyHands. We have found the detailed guidance on choosing appropriate images produced by Cochrane UK’s knowledge brokers to be extremely useful [57] in addition to involving representatives of the potential audience, and others less connected with the work, in the choice and review of images.

### Evaluation

We are mindful of the need for evaluation [58]. Our preference has been to undertake informal evaluation both at the formative and the outcome stage [59]. We are aware that in approaching evaluation in this way we are not able to benefit from objective critique. Nevertheless, as research dissemination often happens beyond the funded stage of a project, formal evaluation can be challenging. We undertake formative evaluation to guide development by identifying optimal content, format and delivery channels during the process of creating content. As described above, an important element of all our work is listening to the potential audience, and formative evaluation takes place through informal discussion via face to face meetings and email. Outcome evaluation, assessing whether an output meets its goals, whether they be behaviours, attitudes, knowledge, skills or intentions is more challenging to undertake informally. Our approach has been to capture metrics on, e.g. blogs and tweets and exploring reasons for some outputs attracting more attention that others, with the intention of incorporating learning into future communication. However, metrics are not easy to analyse and whilst they may guide understanding of the reach of a product, they provide no indication of the extent to which the evidence is subsequently used [60].

### Unexpected benefits

The COVID pandemic has highlighted the vital need for effective science communication that informs and encourages behaviour change at individual, societal and policy levels [61–63]. Reflecting on the process of producing creative communication outputs, we realise that as well as helping us to better understand the context of the research findings and their value for evidence users, we have also observed some unexpected benefits. These include (i) creating interest amongst the general public in understanding and using research, (ii) reducing research waste by enhancing access to existing research findings and engaging those for whom the findings could be useful, (iii) opening up two-way conversation with the end-users of research to discuss their implications and (iv) improving clarity of communication between the research team and the potential end-users of the research.

## Conclusions

In summary, it is widely acknowledged that most stakeholders do not have time to invest in reading large and complex documents. Creative communication tools can be a useful mechanism to improve accessibility to key messages. As well as helping us to better understand the context of the research findings and their value for evidence users, we have also observed some unexpected benefits of the processes of producing creative communication outputs. Furthermore, our experience has highlighted a range of additional benefits of embedding these techniques within our project processes, e.g. opening up two-way conversation with end-users of research to discuss the implications of findings.

## Data Availability

Not applicable.

## References

[1] National institute of Health Research. How to disseminate your research 2019 [Available from: https://www.nihr.ac.uk/documents/how-to-disseminate-your-research/19951#:~:text=Principles%20of%20good%20dissemination,-Stakeholder%20engagement%3A%20Work&text=Format%3A%20Produce%20targeted%20outputs%20that,or%20local%20levels%20as%20appropriate. Accessed 21/04/2021. 2021.

[2] Wallace J, Nwosu B, Clarke M (2012). Barriers to the uptake of evidence from systematic reviews and meta-analyses: a systematic review of decision makers’ perceptions. BMJ Open.

[3] Tricco AC, Cardoso R, Thomas SM, Motiwala S, Sullivan S, Kealey MR (2016). Barriers and facilitators to uptake of systematic reviews by policy makers and health care managers: a scoping review. Implement Sci.

[4] Glenton C, Rosenbaum S, MS. F. Checklist and guidance for disseminating findings from Cochrane intervention reviews: Cochrane; 2019 [Available from: https://training.cochrane.org/sites/training.cochrane.org/files/public/uploads/Checklist%20FINAL%20version%201.1%20April%202020pdf.pdf Accessed 21/04/2021 2021.

[5] Lavis JN, Robertson D, Woodside JM, McLeod CB, Abelson J (2003). How can research organizations more effectively transfer research knowledge to decision makers?. Milbank Q.

[6] Health Foundation. Communicating your research - a toolkit 2017 [Available from: https://www.health.org.uk/publications/communicating-your-research-a-toolkit Accessed 21/04/2021 2021.

[7] Ontario Centre for Excellence for Child and Youth Mental Health. Knowledge Mobilisation Toolkit 2019 [Available from: http://www.kmbtoolkit.ca/ Accessed 21/04/2021 2021.

[8] Oxman AD, Glenton C, Flottorp S, Lewin S, Rosenbaum S, Fretheim A (2020). Development of a checklist for people communicating evidence-based information about the effects of healthcare interventions: a mixed methods study. BMJ Open.

[9] Brownson RC, Eyler AA, Harris JK, Moore JB, Tabak RG (2018). Getting the word out: new approaches for disseminating public health science. J Publ Health Manage Pract.

[10] Jones K, Hollands GJ, Shemilt I, Doyle J, Armstrong R (2016). Planning and implementing a targeted and strategic dissemination plan for a Cochrane review: a case study. J Publ Health (Oxford, England).

[11] Cochrane. Evidently Cochrane 2021 [Available from: https://www.evidentlycochrane.net/ Accessed 21/04/2021 2021.

[12] Cooper A, Gray J, Willson A, Lines C, McCannon J, McHardy K (2015). Exploring the role of communications in quality improvement: a case study of the 1000 lives campaign in NHS Wales. J Commun Healthc.

[13] Male A. ‘The power and influence of illustration’: an invited international keynote lecture 3rd Conference in Illustration and Animation (CONFIA 2015). Braga; 2015. http://repository.falmouth.ac.uk/1580/.

[14] Green MJ, Myers KR (2010). Graphic medicine: use of comics in medical education and patient care. BMJ (Clinical research ed).

[15] Williams IC (2012). Graphic medicine: comics as medical narrative. Medical Human.

[16] Farinella M. The potential of comics in science communication. J Sci Commun. 2018;17. 10.22323/2.17010401.

[17] Baff D (2020). Using Sketchnotes in PhD research and academic practice. Int J Manage Appl Res.

[18] Fernández-Fontecha A, O’Halloran KL, Tan S, Wignell P (2018). A multimodal approach to visual thinking: the scientific sketchnote. Vis Commun.

[19] Dunlap JC, Lowenthal PR (2016). Getting graphic about infographics: design lessons learned from popular infographics. J Visual Literacy.

[20] Agley J, Xiao Y, Thompson EE, Golzarri-Arroyo L (2021). Using infographics to improve trust in science: a randomized pilot test. BMC Res Notes.

[21] Huang S, Martin LJ, Yeh CH, Chin A, Murray H, Sanderson WB (2018). The effect of an infographic promotion on research dissemination and readership: a randomized controlled trial. Cjem.

[22] Joshi M, Gupta L (2021). Preparing infographics for post-publication promotion of research on social media. J Korean Med Sci.

[23] Kunze KN, Vadhera A, Purbey R, Singh H, Kazarian GS, Chahla J. Infographics are more effective at increasing social media attention in comparison with original research articles: an altmetrics-based analysis. Arthroscopy. 2021. 10.1016/j.arthro.2021.03.056. 10.1016/j.arthro.2021.03.056 33838252

[24] Martin LJ, Turnquist A, Groot B, Huang SYM, Kok E, Thoma B (2019). Exploring the role of infographics for summarizing medical literature. Health Profes Educ.

[25] Hunt H, Abbott R, Boddy K, Whear R, Wakely L, Bethel A (2019). “They’ve walked the walk”: a systematic review of quantitative and qualitative evidence for parent-to-parent support for parents of babies in neonatal care. J Neonat Nurs.

[26] Improving communication in multi-disciplinary review teams: reflections on the co-production and use of plain language protocol summaries. 25th Cochrane Colloquium. Edinburgh: Cochrane Database of Systematic Reviews; 2018.

[27] Nunns M, Shaw L, Briscoe S, Thompson Coon J, Hemsley A, McGrath JS (2019). Multicomponent hospital-led interventions to reduce hospital stay for older adults following elective surgery: a systematic review.

[28] Eke H, Hunt H, Ball S, Rogers M, Whear R, Allinson A, et al. Improving continence in children and young people with neurodisability: survey of current NHS practice and systematic review of effectiveness, cost-effectiveness and contextual factors that modify implementation of interventions. Health Technol Assess (Winchester, England). in press. 10.3310/hta25730 34866570

[29] Quintana DS, Heathers JAJ (2021). How podcasts can benefit scientific communities. Trends Cogn Sci.

[30] Thoma B, Goerzen S, Horeczko T, Roland D, Tagg A, Chan TM (2020). An international, interprofessional investigation of the self-reported podcast listening habits of emergency clinicians: a METRIQ study. Cjem.

[31] Moore DA, Nunns M, Shaw L, Rogers M, Walker E, Ford T (2019). Interventions to improve the mental health of children and young people with long-term physical conditions: linked evidence syntheses. Health Technol Assess (Winchester, England).

[32] Roland D, Thoma B, Tagg A, Woods J, Chan TM, Riddell J (2021). What are the real-world podcast-listening habits of medical professionals?. Cureus.

[33] Gatewood J, Monks SL, Singletary CR, Vidrascu E, Moore JB (2020). Social media in public health: strategies to distill, package, and disseminate public health research. J Publ Health Manage Pract.

[34] Yuan S, Besley JC. Understanding science bloggers’ view and approach to strategic communication. Int J Sci Educ Part B. 2021;1-15. 10.1080/21548455.2021.1938741.

[35] Luzón MJ (2013). Public communication of science in blogs: recontextualizing scientific discourse for a diversified audience. Writ Commun.

[36] Whear R, Thompson-Coon J, Rogers M, Abbott RA, Anderson L, Ukoumunne O, et al. Patient-initiated appointment systems for adults with chronic conditions in secondary care. Cochrane Database Syst Rev. 2020;4. 10.1002/14651858.CD010763.pub2. 10.1002/14651858.CD010763.pub2 PMC7144896 32271946

[37] Lavis JN, Permanand G, Oxman AD, Lewin S, Fretheim A (2009). SUPPORT tools for evidence-informed health policymaking (STP) 13: preparing and using policy briefs to support evidence-informed policymaking. Health Res Policy Syst.

[38] Whittam AM, Chow W (2017). An educational board game for learning and teaching burn care: a preliminary evaluation. Scars Burns Heal.

[39] Illingworth S (2020). Creative communication – using poetry and games to generate dialogue between scientists and nonscientists. FEBS Lett.

[40] Abbott R, Orr N, McGill P, Whear R, Bethel A, Garside R (2019). How do “robopets” impact the health and well-being of residents in care homes? A systematic review of qualitative and quantitative evidence. Int J Older People Nursing.

[41] Gröppel-Wegener A, Tracey H, Vigurs K, Black K, Warhurst R (2022). It’s a game of skill: playful learning through board game design’. Organisation studies and human resource management: an Educator’s handbook.

[42] Bartle R (1996). Hearts, clubs, diamonds, spades: players who suit MUDs. J MUD Res.

[43] Oska S, Lerma E, Topf J (2020). A picture is worth a thousand views: a triple crossover trial of visual abstracts to examine their impact on research dissemination. J Med Internet Res.

[44] Hoffberg AS, Huggins J, Cobb A, Forster JE, Bahraini N (2020). Beyond journals-visual abstracts promote wider suicide prevention research dissemination and engagement: a randomized crossover trial. Front Res Metr Analyt.

[45] Bredbenner K, Simon SM (2019). Video abstracts and plain language summaries are more effective than graphical abstracts and published abstracts. PLoS One.

[46] Houts PS, Doak CC, Doak LG, Loscalzo MJ (2006). The role of pictures in improving health communication: a review of research on attention, comprehension, recall, and adherence. Patient Educ Couns.

[47] Cochrane Training. Blogshots 2021 [Available from: https://training.cochrane.org/online-learning/knowledge-translation/how-share-cochrane-evidence/choose-right-dissemination-produ-5 Accessed 16/07/2021 2021.

[48] Grimshaw JM, Eccles MP, Lavis JN, Hill SJ, Squires JE (2012). Knowledge translation of research findings. Implement Sci.

[49] Cochrane. Checklist and guidance for disseminating findings from Cochrane intervention reviews: Cochrane; 2020 [Available from: https://training.cochrane.org/sites/training.cochrane.org/files/public/uploads/Checklist%20FINAL%20version%201.1%20April%202020pdf.pdf Accessed 21/04/2021 2021.

[50] Ashcraft LE, Quinn DA, Brownson RC (2020). Strategies for effective dissemination of research to United States policymakers: a systematic review. Implement Sci.

[51] Rader T, Pardo Pardo J, Stacey D, Ghogomu E, Maxwell LJ, Welch VA (2014). Update of strategies to translate evidence from cochrane musculoskeletal group systematic reviews for use by various audiences. J Rheumatol.

[52] Eljiz K, Greenfield D, Hogden A, Taylor R, Siddiqui N, Agaliotis M (2020). Improving knowledge translation for increased engagement and impact in healthcare. BMJ Open Quality.

[53] Fontaine G, Maheu-Cadotte M-A, Lavallée A, Mailhot T, Rouleau G, Bouix-Picasso J (2019). Communicating science in the digital and social media ecosystem: scoping review and typology of strategies used by health scientists. JMIR Public Health Surveill.

[54] Lavis JN, Lomas J, Hamid M, Sewankambo NK (2006). Assessing country-level efforts to link research to action. Bull World Health Organ.

[55] Wilson MJ, Ramey TL, Donaldson MR, Germain RR, Perkin EK (2016). Communicating science: sending the right message to the right audience. FACETS.

[56] Goldstein CM, Murray EJ, Beard J, Schnoes AM, Wang ML (2020). Science communication in the age of misinformation. Ann Behav Med.

[57] Chapman S, Ryan-Vig S (2020). Choosing images for sharing evidence: a guide. 1.0 ed.

[58] Ziegler R, Hedder IR, Fischer L. Evaluation of science communication: current practices, challenges, and future implications. Frontiers. Communication. 2021;6(73). 10.3389/fcomm.2021.669744.

[59] Wilkinson C, Weitkamp E (2016). Creative research communication.

[60] Langer L, Tripney J, Gough D (2016). The science of using science: researching the use of research evidence in decision-making.

[61] Koerber A (2020). Is it fake news or is it Open Science? Science communication in the COVID-19 pandemic. J Bus Tech Commun.

[62] Bin Naeem S, Kamel Boulos MN. COVID-19 misinformation online and health literacy: a brief overview. Int J Environ Res Public Health. 2021;18(15). 10.3390/ijerph18158091 published Online First: 2021/08/08. 10.3390/ijerph18158091 PMC8345771 34360384

[63] Saitz R, Schwitzer G (2020). Communicating science in the time of a pandemic. JAMA.

